# Path-Integrated Ultrasonic Attenuation Modeling for Concrete with Random Aggregates Based on Modified Waterman–Truell Theory

**DOI:** 10.3390/s26051647

**Published:** 2026-03-05

**Authors:** Haoran Zheng, Chao Lu, Dongjie Zhou, Xuejun Jia, Xiang Lv, Laixin Gao, Guangming Zhang

**Affiliations:** 1 College of Electrical Engineering and Control Science, Nanjing Tech University, Nanjing 211816, China; zhenghaoran@njtech.edu.cn (H.Z.); lc-1206@njtech.edu.cn (C.L.); 202461206144@njtech.edu.cn (D.Z.); 2 China Construction Second Engineering Bureau Co., Ltd., Beijing 100160, China; jxj@njtech.edu.cn (X.J.); jitianyu@njtech.edu.cn (X.L.); 3 School of Mechanical and Electrical Engineering, Chuzhou University, Chuzhou 239000, China; gaolaixin@njtech.edu.cn

**Keywords:** ultrasonic sensing, concrete structures, path-integrated attenuation, aggregate scattering

## Abstract

Ultrasonic sensing is an effective tool for characterizing heterogeneous concrete structures, yet quantitative interpretation of ultrasonic attenuation remains challenging due to aggregate-induced multiple scattering and spatial non-uniformity. This study proposes a path-integrated ultrasonic attenuation modeling framework for concrete with random aggregates. A quasi-one-dimensional discretized wave equation is coupled with a modified version of the Waterman–Truell effective medium theory, in which multiple scattering effects are corrected by incorporating a Percus–Yevick structure factor and a geometric equivalence scheme for non-spherical aggregates. By discretizing the propagation path into locally homogeneous layers, cumulative attenuation is evaluated through explicit path integration, allowing spatial variations in aggregate volume fraction to be captured. Low-frequency ultrasonic transmission experiments (25 kHz) are conducted using serially assembled concrete specimens with controlled aggregate contents. The results reveal pronounced path-dependent attenuation behavior governed by local aggregate distribution. Compared with classical and effective Waterman–Truell models, the proposed approach significantly improves prediction accuracy, achieving a mean absolute percentage error of 7.29%. The framework provides a physically interpretable and experimentally validated method for ultrasonic sensing of heterogeneous concrete, with potential applications in non-destructive evaluation and structural health monitoring of high-end concrete-based engineering structures.

## 1. Introduction

Ultrasonic sensing-based non-destructive evaluation (NDE) techniques have attracted increasing attention in the characterization of cement-based materials and structural health monitoring of concrete structures in recent years [1]. Owing to the strong sensitivity of ultrasonic wave propagation to internal mesostructural features, key information related to material compactness, aggregate distribution, pore structure, and damage state can be obtained without inducing any physical damage to the specimen. As a result, ultrasonic sensing has become an important tool for concrete quality assessment, construction process monitoring, and in-service performance diagnosis, with a clear trend toward online deployment, quantitative interpretation, and model-driven analysis [2,3,4,5].

Unlike homogeneous solids, concrete is a strongly heterogeneous composite consisting of a cementitious matrix, randomly distributed aggregates, and interfacial transition zones (ITZs). The pronounced contrasts in density and acoustic impedance between aggregates and the surrounding matrix, together with the presence of ITZs, inevitably give rise to wave scattering, reflection, and mode conversion during ultrasonic propagation. These mechanisms collectively lead to reduced phase velocity, enhanced amplitude attenuation, and spectral distortion of the received signals [6,7]. At moderate to high aggregate volume fractions, multiple scattering effects become dominant, rendering simplified homogeneous-medium assumptions or conventional empirical ultrasonic indicators inadequate for accurately reflecting the true internal state of concrete [8,9]. This limitation is particularly critical when ultrasonic measurements are used to inversely estimate physical parameters such as mechanical strength or damage degree, and it remains one of the key obstacles restricting the reliability of ultrasonic techniques in engineering applications.

To describe ultrasonic wave propagation in heterogeneous media, effective medium theories and multiple scattering models have been widely introduced into concrete acoustics [10]. Among them, Foldy theory and its extension, the Waterman–Truell (WT) model, provide a fundamental theoretical framework for representing systems of discrete scatterers as equivalent continuous media. By statistically averaging the scattering contributions of aggregates, these models establish quantitative relationships between aggregate volume fraction, scattering characteristics, and the effective attenuation and phase velocity of ultrasonic waves. Such formulations are not only applicable to aggregate-dominated scattering networks but also form the theoretical basis for distinguishing additional scattering induced by internal damage [11]. However, classical WT models generally assume monodisperse spherical scatterers with uncorrelated spatial distributions, which deviates substantially from real concrete systems characterized by non-spherical aggregates, polydispersity, and excluded-volume effects. Consequently, their predictive accuracy deteriorates at moderate aggregate volume fractions.

To overcome these limitations, recent studies have introduced structural-factor corrections, polydisperse scatterer descriptions, and geometric equivalence schemes for non-spherical aggregates to enhance the applicability of effective medium models to real concrete systems. By explicitly accounting for spatial correlations and geometric statistics of aggregates, these modified models provide a more physically consistent description of cumulative multiple scattering effects and improve predictions of attenuation and phase velocity as functions of aggregate content. Nevertheless, at the experimental level, the design of test configurations that can be directly mapped onto such theoretical models, together with systematic validation using controlled parameter sets, remains insufficiently explored. Recent advances attempt to bridge this gap by integrating advanced signal processing techniques [12], high-fidelity numerical simulations [13], and machine-learning-based data interpretation [14].

Despite these advances, most Waterman–Truell-based extensions focus on refining the effective wavenumber formulation itself, such as incorporating spatial correlation, polydispersity, or shape corrections [15]. While these improvements enhance local scattering descriptions, they do not explicitly address cumulative wave propagation in spatially heterogeneous concrete where aggregate volume fraction varies along the ultrasonic path. As a result, the connection between effective medium predictions and measurable transmission attenuation remains indirect.

In contrast, this study extends WT-type modeling by embedding the modified effective wavenumber into a path-integrated propagation framework. This formulation accumulates spatially varying scattering contributions along the propagation direction and establishes direct correspondence between theoretical attenuation and transmission measurements through controlled experimental mapping.

Therefore, the novelty of this work lies not only in modifying the WT formulation, but in bridging effective medium scattering theory and measurable attenuation via path-integrated modeling.

From a sensing perspective, low-frequency ultrasound offers distinct advantages for concrete inspection due to its superior penetration capability and robustness against strong macroscopic heterogeneity. When combined with high-sampling-rate acquisition systems, complete time-domain waveforms can be captured, enabling waveform- and spectrum-based multi-feature analyses. In practical applications, low-frequency ultrasonic arrays coupled with diffusion attenuation and directivity compensation algorithms have demonstrated improved imaging performance in structural blind zones [16], while optimized path selection and inversion strategies have significantly enhanced ultrasonic tomography of internal inclusions [17]. In monitoring time-dependent processes in cement-based materials, novel sensors and multiphysics measurement strategies have enabled precise tracking of early-age material evolution [18,19]. Meanwhile, the focus of research has progressively shifted from sensor hardware toward intelligent interpretation of the acquired ultrasonic data [20].

Motivated by these developments, the present study focuses on ultrasonic attenuation mechanisms dominated by aggregate scattering, with particular emphasis on the following aspects:

1. The influence of aggregate volume fraction on ultrasonic propagation characteristics under a fixed water–cement ratio;
2. The applicability of a modified Waterman–Truell effective medium model to concrete with moderate aggregate volume fractions;
3. The correspondence between path-integrated attenuation predictions and experimental observations.

To this end, a transmission-based ultrasonic testing scheme employing serially assembled specimens is adopted. By systematically controlling aggregate volume fraction and acquiring high signal-to-noise-ratio time-domain signals, the proposed theoretical framework is rigorously validated against experimental data.

The main contributions of this study are summarized as follows:

1. A modified WT model incorporating structural-factor corrections and non-spherical aggregate equivalence is introduced to establish a physically consistent relationship between aggregate volume fraction and effective wavenumber;
2. A path-integration method based on quasi-one-dimensional discretization is developed to accumulate local scattering effects into a global propagation response;
3. A targeted experimental design is implemented to enable direct parameter-level comparison between ultrasonic measurements and theoretical predictions, thereby enhancing the clarity and credibility of model validation.

The remainder of this paper is organized as follows. Section 2 presents the theoretical framework, including discretized modeling of ultrasonic propagation in heterogeneous media, the modified WT effective medium theory, and the path-integration method. Section 3 describes the materials, specimen preparation, ultrasonic testing system, and experimental procedures. Section 4 discusses the experimental results and compares them with theoretical predictions. Section 5 concludes the paper and outlines future research directions.

## 2. Theory

Concrete is a typical heterogeneous composite material, with aggregates randomly distributed in the cementitious matrix, resulting in significant spatial variations in its acoustic properties. Directly simulating ultrasonic wave propagation in such a fully three-dimensional random medium is computationally complex and may obscure the underlying physical mechanisms. Therefore, a physically consistent and computationally feasible modeling framework is required.

The layered discretization represents a statistical homogenization strategy rather than a physical stratification of the material. Effective medium theories, including the Waterman–Truell framework, treat heterogeneous media as ensembles of randomly distributed scatterers whose collective behavior can be represented by averaged local parameters [21]. By choosing a segment thickness larger than individual particle size but smaller than the spatial variation scale of aggregate concentration, each segment may be regarded as locally ergodic while preserving spatial heterogeneity.

Such equivalent-layer representations have been successfully adopted in ultrasonic scattering analyses to model cumulative attenuation through heterogeneous regions without resolving full microstructure, demonstrating reliable correction of scattering-related attenuation predictions [22]. This provides theoretical grounding for mapping spatially varying aggregate distributions into piecewise effective propagation segments.

**Figure 1:**
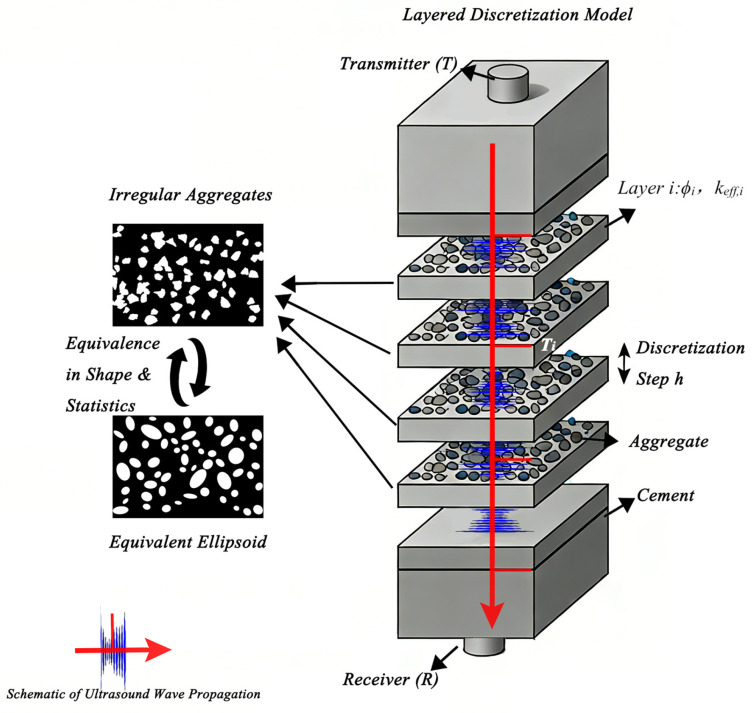
Schematic illustration of the layered discretization framework used for ultrasonic wave propagation modeling in heterogeneous concrete. The propagation path is partitioned into locally homogeneous segments representing spatial variations in aggregate volume fraction and correlation structure. Each segment is assigned effective scattering parameters within the path-integrated formulation. The illustration is conceptual and not to scale; shading and boundaries denote statistical modeling regions rather than physical stratification.

As shown in Figure 1, this study proposes a layered discretization framework, where the specimen is divided into homogeneous layers along the propagation direction (with a characteristic thickness h), and the microstructure within each layer is statistically uniform. Irregular aggregates are replaced by statistically equivalent scatterers, preserving key parameters such as volume fraction $\phi_{i}$ and the corresponding complex wavenumber $k_{eff,i}$. The local propagation effects accumulate along the path, forming the basis for the subsequent path-integration method.

### 2.1. Discretized Wave Equation for Ultrasonic Propagation in Heterogeneous Media

The propagation process of ultrasound in cement paste follows the classic wave dynamics theory. Considering the particle composition of the cement paste, the acoustic parameters are spatially variable. For simplicity, the acoustic pressure field is considered as $p(r,t)=P(r)e^{-i\omega t}$, where P(r) represents the complex pressure amplitude, and the three-dimensional wave propagation equation is established as follows:

(1) $$\nabla^{2}p(r)+k^{2}(r)p(r)=0$$

where $\nabla^{2}=\frac{\partial^{2}}{\partial x^{2}}+\frac{\partial^{2}}{\partial y^{2}}+\frac{\partial^{2}}{\partial z^{2}}$ is the Laplacian operator, and P(r) represents the complex sound pressure (Pa). The wavenumber k(r)=ω/c(r) is the local wavenumber (rad/m), and is a function of the spatial coordinates, reflecting the heterogeneous properties of the cement paste.

Here, ω is the angular frequency (rad/s), and c(r) is the local sound velocity (m/s). The non-uniformity of the cement paste primarily originates from the distribution of the aggregate particles:

(2) $$\begin{array}{c} c(r)=c_{m}+(c_{a}-c_{m})\phi (r)\\ \rho (r)=\rho_{m}+(\rho_{a}-\rho_{m})\phi (r)\\ Z(r)=\rho (r)c(r) \end{array}$$

In this context, c(r) is the local sound velocity, $c_{m}$ and $c_{a}$ represent the sound velocities of cement and aggregate (m/s), respectively. The volume fraction ϕ(r) refers to the aggregate phase, with 0≤ϕ≤1. ρ(r) is the local density (kg/m^3^), and $\rho_{m}$ and $\rho_{a}$ are the densities of cement and aggregate. Z(r) represents the acoustic impedance, which describes the material’s resistance to sound waves.

Directly solving the three-dimensional wave Equation (1) in a random heterogeneous medium poses significant challenges. From a physical perspective, the ultrasound beam generated by the transducer has a certain directionality and concentration. When the transducer’s aperture (approximately 30 mm) is much larger than the characteristic size of the aggregates (5–10 mm) and the specimen’s lateral dimensions are sufficiently large, the energy in the propagation direction (x-axis) dominates, with relatively weak lateral energy diffusion. Based on these physical facts, the beam restriction assumption is introduced: ultrasonic energy mainly propagates along the primary propagation direction (x-axis), and lateral energy diffusion is negligible. The validity of this assumption is supported by the relationship between wavelength and heterogeneity scale as well as by established observations of ultrasonic scattering in concrete. Concrete is known to exhibit strong elastic scattering due to aggregates and pores, yet the coherent transmitted field remains governed by averaged forward propagation behavior when the wavelength exceeds characteristic heterogeneity size. Experimental and modeling studies have shown that effective medium descriptions capture the dominant propagation characteristics in such heterogeneous media through statistical averaging of scatterer distributions [23].

At the operating frequency used here, the ultrasonic wavelength in cementitious materials is on the order of centimeters, exceeding typical aggregate size, which limits strong angular redistribution of energy. Under these conditions, scattering primarily contributes to attenuation of the coherent forward component rather than complete diffusion, making the quasi-one-dimensional cumulative modeling of transmission appropriate. This interpretation is consistent with observations that attenuation in concrete is dominated by scattering interactions with aggregate distributions along the propagation path.

However, when the aggregate volume fraction is too high or the propagation distance is too long, multiple scattering may cause beam divergence, and the accuracy of this assumption will decrease. Therefore, this model is mainly applicable to concrete with a moderate aggregate volume fraction. The mathematical formulation is as follows:

(3) $$\left|\frac{\partial^{2}P}{\partial y^{2}}+\frac{\partial^{2}P}{\partial z^{2}}\right|\ll \left|\frac{\partial^{2}P}{\partial x^{2}}\right|$$

Based on the assumption in (3), a horizontal averaging process is applied to Equation (1). The average sound pressure is defined as:

(4) $$\bar{P}(x)=\frac{1}{S}{\int \int }_{S}P(x,y,z)dydz$$

where S is the cross-sectional area of the wavefront. Substituting Equation (4) into (1) and performing horizontal averaging over the entire equation yields:

(5) $$\frac{1}{S}{\int \int }_{S}\nabla^{2}Pdydz+\frac{1}{S}{\int \int }_{S}k^{2}(r)Pdydz=0$$

The Laplace operator is decomposed into its longitudinal and transverse components: $\nabla^{2}=\frac{\partial^{2}}{\partial x^{2}}+\nabla_{⊥}^{2}$, where $\nabla_{⊥}^{2}=\frac{\partial^{2}}{\partial y^{2}}+\frac{\partial^{2}}{\partial z^{2}}$, and thus, Equation (5) becomes

(6) $$\frac{\partial^{2}}{\partial x^{2}}\left(\frac{1}{S}{\int \int }_{S}Pdydz\right)+\frac{1}{S}{\int \int }_{S}\nabla_{⊥}^{2}Pdydz+\frac{1}{S}{\int \int }_{S}k^{2}(r)Pdydz=0$$

The first term, involving the exchange and integration order, simplifies to: $\frac{d^{2}P}{dx^{2}}$.

Using the divergence theorem, we obtain the transverse term $\frac{1}{S}{\int \int }_{S}\nabla_{⊥}^{2}Pdydz=\frac{1}{S}\oint_{\partial S}\frac{\partial P}{\partial n}dl$, where ∂S is the boundary of the transverse surface. Based on wave energy loss, we assume that the scattering in the transverse direction is weak and that the boundary term $\frac{\partial P}{\partial n}\approx 0$, so this term can be neglected. To transform the three-dimensional non-homogeneous material into a one-dimensional model, we adopt effective medium theory, where we separate the term into a boundary wavenumber $k_{eff}^{2}(x)$ that dominates the longitudinal component, along with a source term for the scattering field $S_{scat}(x)$. Specifically, this approach modifies the model at node 2.3 to ensure the physical closure of the system.

(7) $$\frac{1}{S}{\int \int }_{S}k^{2}(r)Pdydz=k_{eff}^{2}(x)\bar{P}(x)+S_{scat}(x)$$

This yields the simplified one-dimensional wave equation:

(8) $$\frac{d^{2}\bar{P}}{dx^{2}}+k_{eff}^{2}(x)\bar{P}=S_{scat}(x)$$

In this model, $k_{eff}(x)$ is the effective wavenumber, which is a local effective parameter, and $S_{scat}(x)$ represents the effective scattering source term, reflecting the non-homogeneous scattering effects caused by the coarse aggregates.

The simplified one-dimensional wave equation is a continuous form and needs to be solved using the Method of Lines (MOL), which discretizes the spatial domain and transforms the equation into a system of algebraic equations for numerical solution [24]. To perform the frequency-domain solution, the equation for the frequency domain is converted using the Fourier transform, and then the time-domain wave equation is applied for solving. The equation for the one-dimensional wave in the time domain is given by: $\frac{\partial^{2}v}{\partial t^{2}}-c^{2}(x)\frac{\partial^{2}v}{\partial x^{2}}=s(x,t)$, where v(x,t) is the time-dependent acoustic pressure, and s(x,t) represents the time-varying scattering source term.

Divide the x-axis into M evenly spaced points: $x_{i}=ih(i=0,1,...,M,h=l/M)$. Define the point values as approximations to: $v_{i}(t)\approx v(x_{i},t)$, where v is the corresponding time-domain pressure in the region $\bar{P}(x)$. At internal nodes $x_{i}(i=1,...,M-1)$, the spatial second-order derivatives in the 1D wave equation are approximated using central differences.

(9) $$\frac{\partial^{2}v}{\partial x^{2}}{|}_{x_{i}}\approx \frac{v_{i-1}-2v_{i}+v_{i+1}}{h^{2}}+O(\Delta x^{2})$$

Substitute the discretization formula into Equation (8), and combine the source term to obtain the equation for node i:

(10) $$\frac{d^{2}v_{i}}{dt^{2}}-\frac{v_{i-1}-2v_{i}+v_{i+1}}{h^{2}}=q(x_{i},t)$$

In which, $q(x_{i},t)$ represents the combined non-uniform effective scattering source term, and the frequency domain form is related to $k_{eff}(x_{i})$ and $S_{scat}(x_{i})$. The system is concerned with the temporal average of the micro-partial field, which allows the use of the conventional time-averaging method for solving.

This section achieves a horizontal averaging and one-dimensional treatment of the three-dimensional wave equation under wave boundary constraints. Although this process simplifies the three-dimensional random inhomogeneity into local effective parameters, the specific values of the effective wavenumbers still depend on the scattering properties of the materials and their spatial distribution. Therefore, in the Section 2.2, we will return to classic effective medium theories and propose an expression that can be used to compute $k_{eff}$.

### 2.2. Classical Effective Medium Theory: Foldy Approximation and the Waterman–Truell Model

The effective medium theory suggests that the effective medium of scattering systems is equivalent to an average medium, in which Foldy’s multiple scattering theory is the core basis [25]. Foldy’s multiple scattering theory uses statistical averaging methods to treat the scattering field of randomly distributed scatterers as a continuous scattering field. The basic idea is that the total wave field can be expressed as the sum of the incident wave and all scattered waves, and the correlation in scattering space is obtained by approximating the wave field. Under the Foldy approximation, the expression for the effective wavenumber $k_{eff}$ is:

(11) $$k_{\mathrm{eff}}^{2}=k_{0}^{2}+4\pi nf(0)$$

where $k_{m}=\omega /c_{m}$ is the base wavenumber ($c_{m}$ is the speed of sound in the matrix), n is the scatterer density, and f(0) is the single-scattering amplitude in the forward direction (scattering angle is 0°). This model only accounts for single-scattering contributions and is therefore applicable to dilute aggregate systems (i.e., low inclusion volume fraction, ϕ≪1). However, it does not capture multiple scattering interactions that become significant in moderately to densely packed aggregate distributions.

The Waterman–Truell (WT) model introduces a second-order scattering correction based on the Foldy approximation to improve the accuracy of dense systems [26]. The standard form is:

(12) $$k_{\mathrm{eff}}^{2}=k_{m}^{2}+4\pi nf(0)+\frac{{\left(4\pi nf(0)\right)}^{2}}{k_{m}^{2}}$$

Physically, the right-hand three terms correspond to the following. $k_{m}^{2}$: the contribution of the base material’s wavenumber. ${\left(4\pi nf(0)\right)}^{2}$: the single-scattering effect. ${\left(4\pi nf(0)\right)}^{2}/k_{m}^{2}$: the second-order scattering correction, representing the mutual interaction of scattered waves in scattering space.

The WT model is more accurate for intermediate volume fractions (ϕ≈0.1−0.3), but it assumes the scatterers are single spherical particles and have uniform distribution in space. This assumption does not adequately represent the non-spherical morphology, multiple scattering interactions, and random spatial distribution of aggregates in heterogeneous concrete. Therefore, further development is needed by integrating structural factors and non-spherical scatterer effects.

### 2.3. Modified WT Model Incorporating Structural Factor and Non-Spherical Aggregate Equivalence

The equivalent-sphere representation is adopted as a statistical scattering approximation rather than a geometric substitution. In heterogeneous media, effective medium and multiple scattering theories commonly employ simplified inclusion geometries when orientations are random, since macroscopic effective parameters are primarily governed by concentration and size statistics rather than detailed morphology. This principle underlies classical treatments of wave propagation in particulate composites with randomly distributed spherical inclusions used to determine effective moduli and attenuation behavior [27].

For ultrasonic propagation in concrete, numerical and experimental studies further indicate that coherent wave parameters are more sensitive to inclusion concentration and distribution than to aggregate shape, with shape effects playing a secondary role under typical wavelength–particle size regimes [28]. Therefore, matching statistical descriptors such as volume fraction and correlation distance preserves the leading-order forward-scattering contribution entering the WT formulation, justifying the approximation for effective medium prediction accuracy.

In ultrasonic concrete testing, the classical WT model, due to its assumption that scatterers are monodisperse spheres and its neglect of shape effects, struggles to accurately describe wave propagation in heterogeneous media. This section modifies the WT model based on the Percus–Yevick (PY) hard-sphere theory and the equivalent non-spherical aggregate method. The core of the modification includes two aspects. First, the introduction of an analytical solution for the structural factor under the PY approximation to capture multiple scattering effects in dense systems; second, by eliminating the volume-distance distribution function matching, non-spherical aggregates are equivalently treated as a polydisperse sphere system, resolving the errors caused by shape variations, and ultimately providing an explicit expression for the local effective wavenumber.

For a polydisperse hard-sphere system, the structure factor S(q) is a key parameter that influences the effective wavenumber [29]. The structure factor formulation adopted here originates from statistical-mechanics descriptions of disordered particulate systems. Its use is not material-specific but reflects spatial correlation among scatterers, which directly governs coherent multiple scattering behavior in effective medium theories. Since concrete aggregates form a dense random packing analogous to hard-particle assemblies, the same statistical descriptors can be employed to characterize correlation effects entering the WT-based effective wavenumber formulation. In the limit as q→0, S(0) represents the compressibility of the system, which is directly related to the volume fraction ϕ. Based on the PY theory, for the distribution of the system’s radii (with the number density function $n(a)\propto e^{-a/\langle a\rangle }$, where $\langle a\rangle$ is the mean radius), S(q) simplifies to

(13) $$S(q)={\left(1-\phi \right)}^{2}\frac{{\left(1+x^{2}\right)}^{3}}{5+x^{2}}\frac{P_{2}(x^{2})}{Q_{4}(x^{2})}$$

where $x=2q\langle a\rangle$, ϕ is the volume fraction, and $P_{2}$ and $Q_{4}$ are polynomial terms:

(14) $$\begin{array}{c} P_{2}(Z)=5+4\phi^{2}+2(3-5\phi +3\phi^{2})Z+{\left(1-\phi \right)}^{2}Z^{2}\\ Q_{4}(Z)={\left(1+2\phi \right)}^{2}+4\left(1+5\phi^{2}\right)Z+2(3-8\phi +9\phi^{2}-2\phi^{3})Z^{2}+4{\left(1-\phi \right)}^{4}Z^{3}+{\left(1-\phi \right)}^{4}Z^{4} \end{array}$$

When q=0(i.e., x=0), the analytical expression for S(0) is obtained as:

(15) $$S(0)=\frac{{\left(1-\phi \right)}^{2}(5+4\phi^{2})}{5{\left(1+2\phi \right)}^{2}}$$

The equation shows that when ϕ≪1, S(0)≈1, which satisfies the dilute system limit. As ϕ increases, S(0) deviates from 1, reflecting the compressibility variation caused by repulsive forces. This modification corrects the classical WT model, where the volume fraction and the effective wavenumber are related in a simplistic manner.

For non-spherical aggregate particles, analytical structure factors are generally not available. Therefore, numerical estimation methods based on equivalent exclusion-volume statistics are adopted. These approaches approximate irregular particle geometries through parameters such as volume and surface area that determine nearest-neighbor separation, allowing structure-factor evaluation without explicit shape resolution [30].

Although these numerical formulations were originally developed for diffraction and particulate-scattering studies, they describe geometric packing constraints independent of material class. In the present work, they are employed solely to capture spatial correlation effects of aggregates within the ultrasonic scattering model.

The total distance distribution function $p_{\mathrm{tot}}(r)$ can be solved as:

(16) $$p_{\mathrm{tot}}(r)=p_{1}(r)-\eta p_{\mathrm{excl}}(r)+\eta p_{\mathrm{struct}}(r)$$

In which, $p_{1}(r)$ is the single-particle distance distribution function, η is the volume fraction, $p_{\mathrm{excl}}(r)$ is the volume exclusion term, and $p_{\mathrm{struct}}(r)$ represents the structural term. The exclusion function $p_{\mathrm{excl}}(r)$ can be accurately calculated using Monte Carlo simulations.

The exclusion term for polydisperse systems $p_{\mathrm{excl}}(r)$ can be expressed by the following equation:

(17) $$p_{\mathrm{exel}}(r)=\int_{0}^{\infty }f(R_{j})\int_{0}^{\infty }f(R_{i})\int_{0}^{R_{i}+R_{j}}\frac{a^{2}}{\langle V\rangle }p_{x}(r,a,R_{i},R_{j})dadR_{i}dR_{j}$$

In which $p_{x}$ is the distance distribution function between two spheres (with radii $R_{i}$, $R_{j}$ and the center-to-center distance a), f(R) is the radius distribution function. By adjusting the parameters of the spherical radius distribution f(R), the system’s $p_{\mathrm{excl}}(r)$ can be modified to account for non-spherical aggregate materials.

Using a statistical fitting approach based on two-dimensional projection images, several key parameters can be measured, including the projected area A, perimeter P, and major/minor axis lengths $L_{major}$, $L_{minor}$. These measurements are used to compute the three-dimensional characteristic parameters through statistical relations.

(18) $$V_{\mathrm{est}}=k_{V}\cdot A^{3/2},\;S_{\mathrm{est}}=k_{S}\cdot P^{2}$$

The correction factors $k_{V}$ and $k_{S}$ are determined through standard experimental calibration.

The exclusion function $p_{\mathrm{excl}}^{\left(\mathrm{actual}\right)}(r)$ for the actual aggregate system is calculated by using Monte Carlo simulations. Under boundary conditions with randomly distributed aggregate particles, the distribution characteristics of the distance between the centers of neighboring aggregate particles are computed, and the optimization model is established to minimize:

(19) $$\underset{\langle R\rangle ,s}{\min}\int_{0}^{\infty }{\left[p_{\mathrm{excl}}^{\left(\mathrm{actual}\right)}(r)-p_{\mathrm{excl}}^{\left(\mathrm{sphere}\right)}(r;\langle R\rangle ,s)\right]}^{2}w(r)dr$$

where $p_{\mathrm{excl}}^{\left(\mathrm{actual}\right)}(r)$ is fully determined by three-dimensional parameters, and $p_{\mathrm{excl}}^{\left(\mathrm{sphere}\right)}(r;\langle R\rangle ,s)$ is the theoretical exclusion function for an effective spherical system. The parameters $\langle R\rangle$ and s are calibrated, with w(r) being the weight function sensitive to acoustic scattering. The distance function is adjusted to ensure good acoustic efficiency. The Schulz distribution parameters for the ellipsoidal shape (with the major axis a, and aspect ratio ε) are given by:

(20) $$\langle R(\varepsilon )\rangle =a[1+c_{R}\ln(\varepsilon )],\;s(\varepsilon )=c_{s}(1-\varepsilon )$$

The constants $c_{R}$ and $c_{s}$ are given by the following.

For ε<1, $c_{R}=0.375$, $c_{s}=0.348$;

For ε>1, $c_{R}=-0.678$, $c_{s}=-0.264$.

This method eliminates shape dependence through statistical corrections, allowing complex aggregate materials to be approximated by spherical systems based on the PY solution.

With this correction, the effective wavenumber $k_{eff}$ in the WT model (Equation (12)) is updated as:

(21) $$k_{\mathrm{eff}}^{2}=k_{m}^{2}\left[1+\frac{4\pi nf_{\mathrm{eff}}(0)}{k_{m}^{2}}+{\left(\frac{4\pi nf_{\mathrm{eff}}(0)}{k_{m}^{2}}\right)}^{2}S(0)\right]$$

In which $k_{m}=\omega /c_{m}$ is the base wavenumber, n is the scatterer density, S(0) is the structure factor, and $f_{\mathrm{eff}}(0)$ is the effective forward scattering function. For non-spherical aggregate materials, $f_{\mathrm{eff}}(0)$ is calculated by using the equivalent spherical distribution.

(22) $$f_{\mathrm{eff}}(0)=\int_{0}^{\infty }f(R,0)p(R;{\langle R\rangle }^{*},s^{*})dR$$

In which $p(R;{\langle R\rangle }^{*},s^{*})$ is based on the effective parameters ${\langle R\rangle }^{*}$ and $s^{*}$ from the Schulz distribution function:

(23) $$p(R;\langle R\rangle ,s)={\left(\frac{z+1}{\langle R\rangle }\right)}^{z+1}R^{z}\exp\left[-\left(\frac{z+1}{\langle R\rangle }\right)R\right]/\Gamma (z+1)$$

In which ${\langle R\rangle }^{*}$ and $s^{*}$ are obtained by optimizing the exclusion volume distribution to the effective spherical system’s mean radius and dispersion. The shape parameter z and the dispersion s satisfy $s={\left(z+1\right)}^{-1/2}$, where z is determined by the distribution of actual aggregate material size, and s becomes larger as the particle size distribution widens. The Gamma function Γ(z+1) is used in the calculation.

The number density $n=\phi /V_{p}$ and the volume $V_{p}=4\pi {\langle R\rangle }^{3}/3$ are substituted into the corrected WT model (21):

(24) $$k_{\mathrm{eff}}^{2}=k_{m}^{2}\left[1+\frac{4\pi \phi f_{\mathrm{eff}}(0)}{k_{m}^{2}(4\pi {\langle R\rangle }^{3}/3)}+{\left(\frac{4\pi \phi f_{\mathrm{eff}}(0)}{k_{m}^{2}(4\pi {\langle R\rangle }^{3}/3)}\right)}^{2}S(0)\right]$$

The simplified expression is:

(25) $$k_{\mathrm{eff}}^{2}=k_{m}^{2}\left[1+\frac{3\phi f_{\mathrm{eff}}(0)}{k_{m}^{2}{\langle R\rangle }^{3}}+{\left(\frac{3\phi f_{\mathrm{eff}}(0)}{k_{m}^{2}{\langle R\rangle }^{3}}\right)}^{2}S(0)\right]$$

Given $A=3f_{\mathrm{eff}}(0)/k_{m}^{2}{\langle R\rangle }^{3}$ and $B=A^{2}$, the effective wavenumber in the local region is simplified as:

(26) $$k_{\mathrm{eff}}^{2}=k_{m}^{2}\left[\begin{array}{c} 1+A\phi +B\phi^{2}S(0) \end{array}\right]$$

For the actual aggregate system, where the size distribution follows the Schulz distribution, the precise PY solution is simplified using a truncation method, and the following approximation is obtained:

(27) $$S(0)\approx \frac{{\left(1-\phi \right)}^{4}}{{\left(1+2\phi \right)}^{2}}\left[1+\frac{s^{2}\phi (3-2\phi )}{2{\left(1-\phi \right)}^{2}}\right]$$

where s is the dispersion, and ϕ is the aggregate volume fraction.

**Figure 2:**
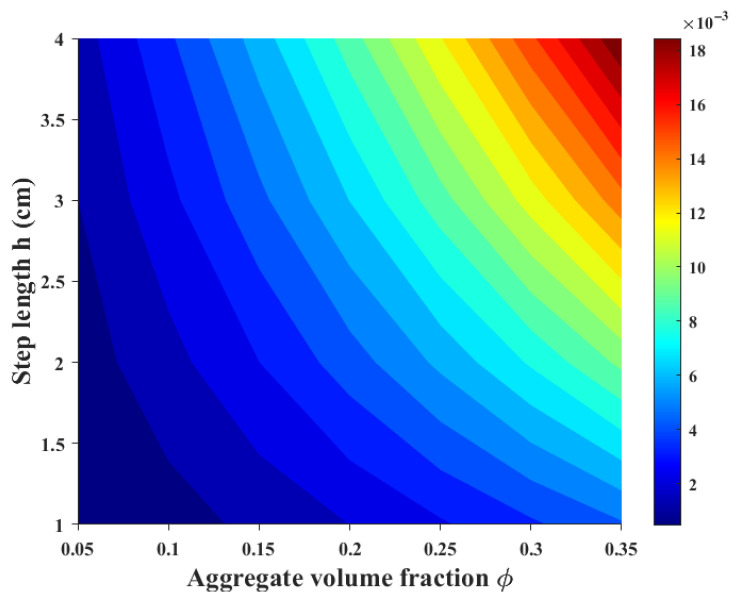
Predicted local attenuation contribution as a function of aggregate volume fraction and discretization step size. Results are computed using the proposed path-integrated effective wavenumber formulation, where attenuation represents the incremental scattering loss associated with each locally homogeneous segment. All parameters except those shown are held constant as defined in the model formulation, and simulations correspond to the 25 kHz ultrasonic excitation used in this study.

As shown in Figure 2, the attenuation of the scattering contribution increases with the aggregate volume fraction ϕ, indicating that aggregate materials with higher content have stronger scattering effects. Additionally, it is observed that the attenuation is linearly related to the scattering step length h, which is consistent with the path integral method used in this study.

The modified WT model established in this section introduces the Percus–Yevick structural factor and the non-spherical aggregate approximation, effectively addressing the limitations of the classical model in ultrasound scattering analysis of heterogeneous concrete. This model not only preserves the theoretical foundation of the WT model but also incorporates the multiple scattering effects in dense systems through the structural factor modification, providing a solid theoretical basis for the subsequent path integral method.

### 2.4. Path-Integration Method Based on the Discretized Wave Equation

Based on the framework established in Section 2.1, a complete path integral method is developed. This method transforms the wave propagation process into a calculation of the position of scattering points in space, providing a feasible solution for high-efficiency ultrasonic testing in concrete.

From the scattering results in Section 2.1, considering the frequency domain, the propagation function $v_{i}(t)=V_{i}e^{-i\omega t}$ is substituted into the scattering Equation (10), and the transformation to the frequency domain is as follows:

(28) $$-\omega^{2}V_{i}-\frac{V_{i-1}-2V_{i}+V_{i+1}}{h^{2}}=Q(x_{i})$$

This is simplified as:

(29) $$\frac{V_{i-1}-2V_{i}+V_{i+1}}{h^{2}}+k^{2}(x_{i})V_{i}=-Q(x_{i})$$

In which $k^{2}(x_{i})=\omega^{2}/c^{2}(x_{i})$, and $Q(x_{i})$ is the frequency domain source term.

First, consider the homogeneous source-free case ($Q(x_{i})=0$), simplifying the equation as:

(30) $$V_{i-1}-2V_{i}+V_{i+1}+k^{2}(x_{i})h^{2}V_{i}=0$$

Next, assume that the wave propagation within each small interval ($\left[x_{i-1},x_{i}\right]$) can be approximated as uniform within the region. Define the local transmission coefficient $P_{i}$ as:

(31) $$V_{i}=P_{i}\cdot V_{i-1}$$

where $P_{i}$ represents the transmission coefficient from point i−1 to point i.

Substituting $V_{i}=P_{i}\cdot V_{i-1}$ and $V_{i+1}=P_{i+1}V_{i}=P_{i+1}P_{i}V_{i-1}$ into the difference Equation (30), we get:

(32) $$V_{i-1}-2P_{i}V_{i-1}+P_{i+1}P_{i}V_{i-1}+k^{2}(x_{i})h^{2}P_{i}V_{i-1}=0$$

Eliminating $V_{i-1}$, we arrive at the equation for $P_{i}$:

(33) $$P_{i}(P_{i+1}-2+k^{2}(x_{i})h^{2})=-1$$

For scattering step sizes h that are small compared to the size of the aggregate material, under the condition that each scattering layer can be approximated as having uniform medium, we can approximate $P_{i+1}\approx P_{i}$. This leads to:

(34) $$P_{i}^{2}-(2-k^{2}(x_{i})h^{2})P_{i}+1=0$$

The solution is:

(35) $$P_{i}=\frac{2-k^{2}(x_{i})h^{2}\pm \sqrt{{\left(2-k^{2}(x_{i})h^{2}\right)}^{2}-4}}{2}$$

Under the condition of the continuous limit, the transmission between adjacent scattering layers can be expanded as:

(36) $$P_{i}\approx \exp\left(ik(x_{i})h-\frac{k^{2}(x_{i})h^{2}}{2}+\ldots \right)$$

By retaining the leading term, the approximate solution is:

(37) $$P_{i}\approx \exp(ik(x_{i})h)$$

In the non-uniform medium, the sound resistance changes cause the reflection and transmission at the interface. Based on the wavefront limitation assumption (mainly along the x-axis), the angle of incidence at the interface is small, so the transmission coefficient at the first interface can be defined as:

(38) $$T_{i}=\frac{2Z_{i}}{Z_{i-1}+Z_{i}}$$

where $Z_{i}=\rho_{i}c_{i}$ is the acoustic impedance, $\rho_{i}$ is the density of the medium, and $c_{i}$ is the speed of sound in the medium. This equation gives the transmission coefficient for normal incidence.

This method is a good approximation for wave transmission across an infinite planar boundary. Based on the wavefront limitation assumption in Section 2.1, it accounts for the main portion of the energy loss due to sound impedance mismatches at the interface. Considering both transmission and interface scattering within the medium, the complete diffusion relation is:

(39) $$V_{i}=T_{i}\cdot \exp(ik(x_{i})h)\cdot V_{i-1}$$

From i=1 to i=N, the recursion is:

(40) $$\begin{array}{c} V_{1}=T_{1}\exp(ik(x_{1})h)V_{0}\\ V_{2}=T_{2}\exp(ik(x_{2})h)V_{1}=T_{2}T_{1}\exp(ik(x_{2})h+ik(x_{1})h)V_{0} \end{array}$$

For the recursion up to the N-th point:

(41) $$V_{N}=V_{0}\cdot \prod_{n=1}^{N}T_{n}\cdot \exp\left(i\sum_{n=1}^{N}k_{\mathrm{eff}}[\phi (x_{n})]h\right)$$

where $V_{0}$ is the initial vibration amplitude, $\prod_{n=1}^{N}T_{n}$ is the transmission coefficient for each interface, and $k_{\mathrm{eff}}[\phi (x_{n})]$ is the effective wavenumber. Based on the corrected WT model in Section 2.3 (Equation (26)), h is the step length.

**Figure 3:**
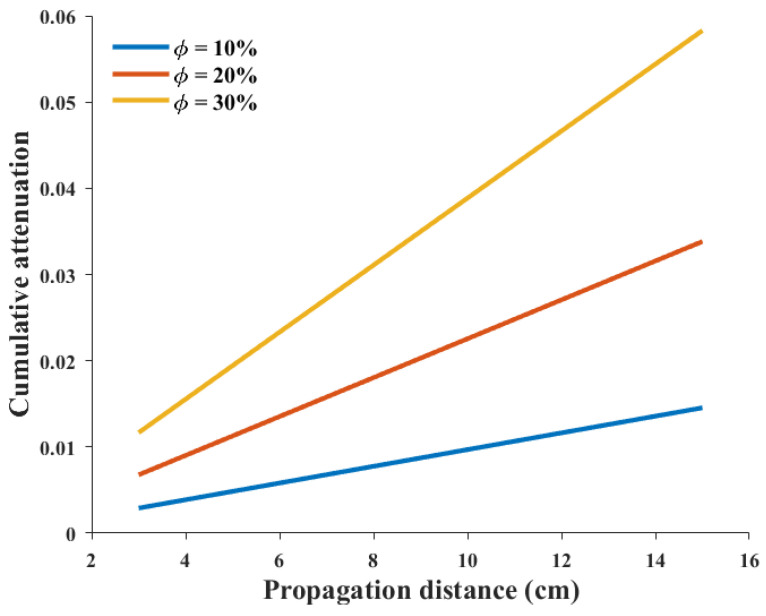
Path-integrated attenuation versus propagation distance for different aggregate volume fractions. Curves are obtained from the proposed path-integrated scattering model, in which attenuation reflects cumulative multiple scattering interactions and progressive redistribution of coherent wave energy. All parameters except aggregate content are fixed as defined in the model formulation, and simulations correspond to the 25 kHz ultrasonic excitation used throughout this work.

As shown in Figure 3, the scattering-induced attenuation increases with propagation distance, indicating progressive loss of coherent wave energy due to cumulative multiple scattering interactions. This behavior is consistent with theoretical expectations for heterogeneous media, where attenuation reflects integrated scattering strength along the propagation path rather than local interaction alone.

Based on the standard wave propagation scattering formalism, a complete path integral method is developed in this section. This method transforms the continuous transmission path scattering into a series of small line segments and applies local approximations at each segment, where the local medium is approximated and the transmission coefficients and scattering locations are aggregated.

## 3. Materials and Methods

This study is based on the modified effective medium theory and the path integral modeling framework, using ultrasonic transmission experiments to quantitatively investigate the ultrasonic propagation characteristics of concrete samples under different aggregate volume fraction conditions. This section describes the materials composition, sample preparation, ultrasonic testing system, experimental procedure, and data processing methods.

### 3.1. Materials and Specimen Preparation

The experiment uses P·O 42.5 ordinary Portland cement (Jiuchang Building Materials Business Department, Shandong, China), with its chemical composition conforming to the GB/T 176-2017 standard [31], as shown in Table 1. The aggregates selected are natural crushed stones, which are cleaned and dried before use. The density of the aggregates is 2600 kg/m^3^, and the particle size range of the aggregates is controlled between 5 and 10 mm to meet the applicable conditions for ultrasonic beam scale and scattering theory assumptions.

To eliminate the influence of changes in the water-to-binder ratio on flowability and acoustic properties, this study fixes the water-to-binder ratio at 0.40. Based on this, three groups of concrete samples with different aggregate volume fractions are prepared, with values of ϕ=0.10,0.20,0.30, as shown in Table 2. The study investigates the impact of variations in aggregate content on ultrasonic scattering and attenuation.

**Figure 4:**
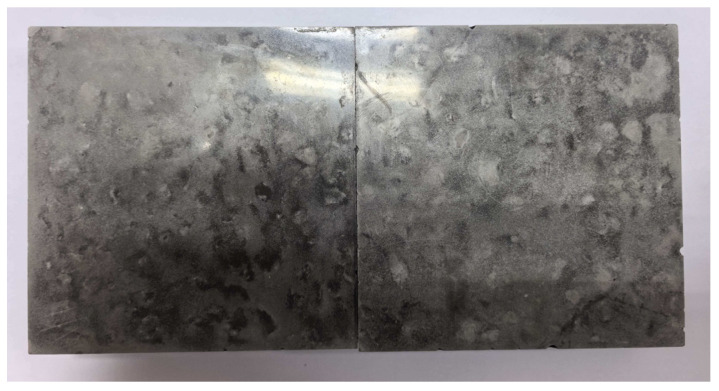
Concrete specimen after surface grinding treatment prior to ultrasonic measurements. Surface preparation improves acoustic coupling consistency between the transducer and specimen and minimizes additional interface-induced scattering. The photograph shows the representative specimen condition used for transmission experiments; dimensions and preparation procedures are described in Section 3.

The mixture is prepared in a constant temperature environment (25 ± 1 °C). First, the cement and aggregates are dry-mixed for 1 min according to the designed ratio. Then, a specified amount of water is added, and the mixture is stirred for an additional 3 min to ensure uniform dispersion of the aggregates in the paste. After mixing, the mixture is poured into a silicone mold with internal dimensions of 100 mm × 100 mm × 30 mm, and any large air bubbles are removed through slight vibration. After leveling the surface, the sample is placed in a standard curing environment (temperature 20 ± 2 °C, relative humidity ≥ 95%) for curing until the required test age is reached. The sample is shown in Figure 4.

Due to the limited optical contrast between the aggregates and cement paste on the surface of the sample, directly identifying the aggregate distribution from the hardened specimen is difficult. Therefore, this study uses a supplementary two-dimensional statistical construction method to characterize the spatial random distribution features of aggregates corresponding to different volume fractions.

In the experiment, aggregates with a particle size distribution consistent with the actual test are randomly arranged on a two-dimensional plane. The projection area fraction is controlled to reach the preset level. The generated aggregate arrangements are imaged and binarized to obtain two-dimensional aggregate projection images. These constructed projection images are independent of the experimental specimens and are used as statistically representative random media for subsequent structural analysis.

### 3.2. Specimen Assembly and Fixture Design

To meet the theoretical assumptions of “long propagation path, multilayer cumulative attenuation” in the transmission method experiment, a multi-sample series testing scheme is adopted. During testing, five sample blocks are arranged in sequence along the ultrasonic propagation direction to form an equivalent propagation path. The surfaces of the sample blocks are polished, and an ultrasonic coupling agent is evenly applied between adjacent sample blocks to reduce reflection and additional loss caused by the air layer at the interfaces, ensuring that the interlayer interfaces closely approach the ideal contact conditions in theory.

**Figure 5:**
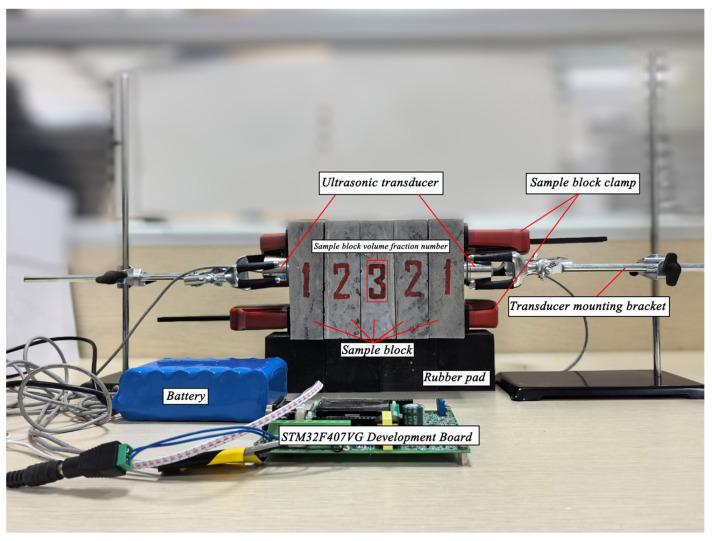
Schematic diagram of the ultrasonic experimental platform used for transmission measurements. The illustration shows the relative placement of transmitting and receiving transducers, specimen positioning, and signal acquisition pathway. This configuration defines the measurement geometry employed for attenuation evaluation, with detailed parameters provided in Section 3.

As shown in Figure 5, the sample combination is fixed using a fixture. The fixture adopts a rigid structure to ensure the stability of the sample’s position and alignment of the axis during the testing process, thereby reducing measurement errors caused by eccentricity or poor contact. This combination method physically corresponds to the discrete multilayer homogeneous medium along the propagation direction in the theoretical model, which is conducive to experimental verification of the path integral method.

### 3.3. Ultrasonic Testing System and Procedure

The ultrasonic testing is arranged using the transmission method. Both the transmitter and receiver use low-frequency piezoelectric ceramic transducers (model: DYW-H25-200CTY) (Dayu Electronics Technology Co., Ltd., Fujian, China) with a center frequency of 25 kHz to enhance the penetration of ultrasonic waves in concrete and improve sensitivity to aggregate scattering effects. The transducers are placed directly on both ends of the sample combination, and the contact areas are coated with a coupling agent to reduce energy loss caused by impedance mismatch at the interface.

The excitation signal is generated by a signal generator module, producing a finite-period sine pulse, which is amplified and applied to the transmitting transducer. The received signal is pre-amplified and then input to the data acquisition system for recording. The sampling frequency is set at 1 MHz to ensure accurate capture of the low-frequency ultrasonic signal’s time-domain waveform.

Before formal testing, the system is first calibrated under no-load and baseline conditions to eliminate the effects of electronic noise and system delay. During testing, the ambient temperature is kept stable to avoid interference with sound speed and material parameters due to temperature fluctuations. For each aggregate volume fraction combination, multiple tests are repeated, and the average results are taken to improve data stability and reliability.

### 3.4. Signal Processing and Acoustic Parameter Extraction

The collected raw ultrasonic signals are first corrected for the DC component to eliminate hardware zero drift. The Hilbert transform is then applied to extract the instantaneous amplitude envelope of the signal, which characterizes the energy attenuation in the sample combination. To suppress random noise interference, the envelope curve is smoothed.

Based on the physical model of exponential amplitude decay in wave propagation through heterogeneous media, the natural logarithm of the envelope amplitude is taken within the stable attenuation region and linearized against propagation distance. The slope of the fit corresponds to the equivalent attenuation coefficient, which represents the energy loss per unit propagation distance due to scattering and absorption.

This attenuation coefficient directly corresponds to the cumulative attenuation predicted by the theoretical model, based on the modified WT model and path integral method, thus providing experimental validation for the effectiveness of the theoretical model under different aggregate volume fraction conditions.

## 4. Results and Discussion

### 4.1. Equivalent Aggregate Statistical Representation and Its Rationality Analysis

The experimental subjects in this study are three-dimensional concrete samples with coarse aggregate volume fractions of 10%, 20%, and 30%. In the equivalent modeling and statistical analysis based on two-dimensional images, the two-dimensional aggregate projection area fractions corresponding to the volume fractions were not directly used as 10%, 20%, and 30%. Instead, 15%, 25%, and 35% were chosen as the corresponding two-dimensional aggregate projection area fractions.

This approach arises from the inherent geometric bias in two-dimensional sectioning or projection statistics of a three-dimensional random particle system. For three-dimensional aggregates with random orientations and distributions, when they are sectioned or projected onto a two-dimensional plane, the probability of larger particles appearing in the plane is significantly higher than their volumetric proportion, resulting in a systematic overestimation of the two-dimensional projection area fraction in statistical averages compared to the corresponding three-dimensional volume fraction. This phenomenon is particularly pronounced under medium to high volume fraction conditions.

Therefore, if the area fractions of 10%, 20%, and 30% are directly used in the two-dimensional equivalent analysis, it would inevitably underestimate the spatial occupation of the aggregates in the two-dimensional statistical plane and weaken the correspondence between the subsequent statistical distribution function and the actual material structure. Based on the above considerations, this study selects 15%, 25%, and 35% as the two-dimensional projection area fractions corresponding to the experimental volume fraction conditions, to more reasonably represent the spatial distribution characteristics of the aggregates in the two-dimensional statistical sense.

To evaluate the effectiveness of the model in describing the real aggregate system in concrete, this study constructs three statistical functions based on two-dimensional and three-dimensional models: p(r), g(r), and S(q), which describe the spatial structure of aggregates in concrete. These functions characterize the aggregate distribution and provide a comprehensive statistical representation.

1. *p*(*r*) represents the radial distribution function, which is used to describe the spatial correlation of aggregate particles in the material. This function is mainly reflective of the size, shape, and scattering properties of the individual aggregates, and is commonly used as a statistical quantity to evaluate the alignment of aggregates in real materials.
2. *g*(*r*) is the pair correlation function, which describes the correlation between the distances of aggregate particles in the material. It is more sensitive to the distance between particles and the structural configuration of the aggregate system. Compared to *p*(*r*), *g*(*r*) is more sensitive to the structural features.
3. *S*(*q*) is the structure factor, which is the response of *g*(*r*) in reciprocal space, used to describe the system at different spatial frequencies *q*. The structure factor is important for characterizing the system’s density at large scales. At low-frequency regions *q* → 0, it corresponds to the density of the system. In the context of ultrasound scattering and attenuation theory, this factor has a direct physical meaning and is used to control the effective wave vector and the macroscopic reduction of scattering at low frequencies.

**Figure 6:**
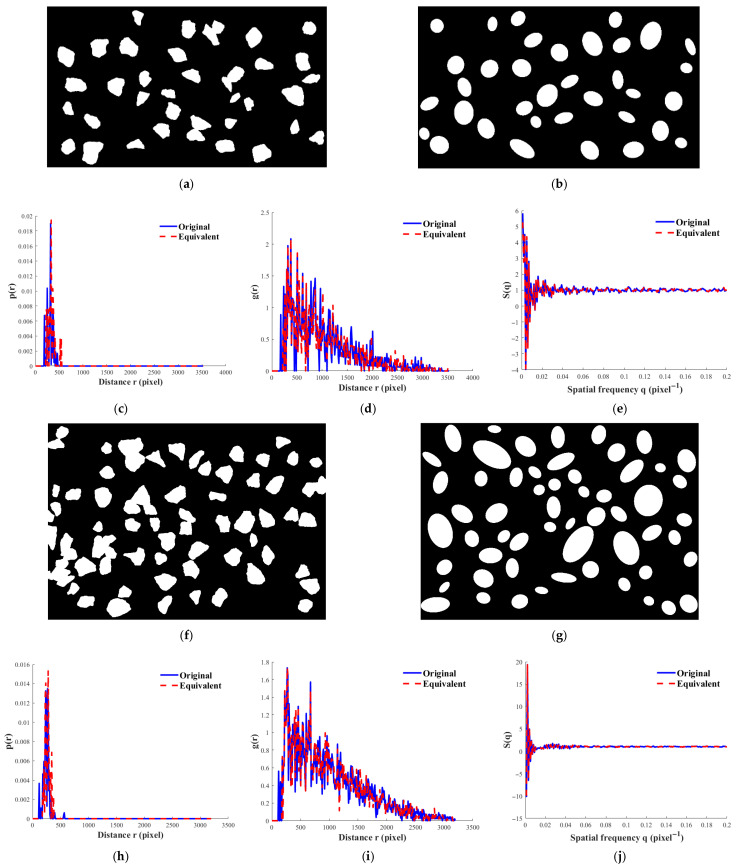

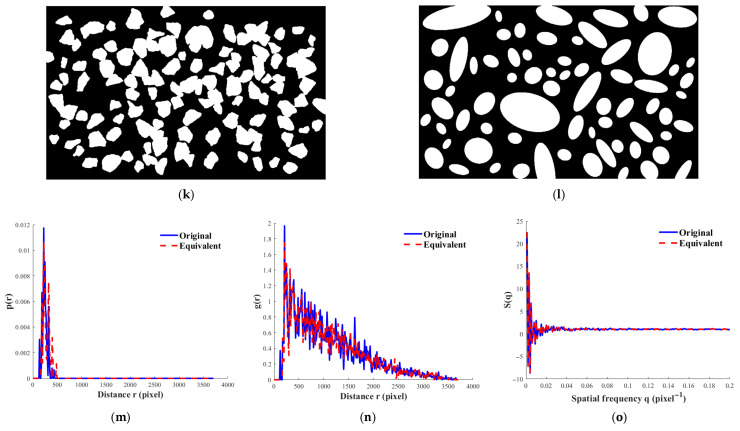
Comparison between real aggregate projections, equivalent ellipsoidal projections, and corresponding statistical descriptors at different projected area fractions. For each fraction, aggregate distributions are shown together with statistical measures used to validate geometric equivalence between representations. (**a**–**e**) Results for 15% projected area fraction: (**a**) original aggregate projection, (**b**) equivalent ellipsoidal projection, (**c**) pair-distance distribution p(r), (**d**) radial distribution function g(r), and (**e**) structure factor S(q). (**f**–**j**) Corresponding results for 25% projected area fraction. (**k**–**o**) Corresponding results for 35% projected area fraction. Projected area fraction denotes the two-dimensional statistical analog of aggregate volume fraction used for model validation.

Figure 6 shows the comparison of the statistical results for real aggregate projections and the corresponding p(r), g(r), and S(q) under three different two-dimensional projection area fractions (15%, 25%, and 35%). Note that r and q represent the two-dimensional projection area’s spatial distance and spatial frequency, respectively. To ensure statistical consistency, elemental unit representations are used. The specific volume fraction is given in Table 3.

The comparison results of p(r) show that under the three projection area fraction conditions, the equivalent polyhedral model can accurately reproduce the geometric statistical characteristics of the actual aggregate system. As the projection area fraction increases from 15% to 35%, the root mean square error of p(r) gradually decreases, indicating that under higher aggregate content conditions, the equivalent geometric model’s statistical approximation ability to the individual structure improves further.

This trend suggests that as the amount of aggregate increases and the statistical sample becomes more comprehensive, the equivalent polyhedral model can more stably represent the geometric features of low real aggregates in the average sense.

Compared to p(r), g(r) is more sensitive to the spatial arrangement between aggregates, with its root mean square error overall higher than that of p(r). This result indicates that although the equivalent polyhedral model cannot completely replicate the local distribution details of real aggregates in the strict sense, its ability to describe the mid-range statistical structure significantly improves as the aggregate fraction increases. Especially when the fraction exceeds 25%, the overall trend of g(r) can more accurately reflect the average spatial correlation characteristics of the real aggregate system.

In the inverse space statistics, the S(q) curve under the three projection area conditions shows good consistency. The effective model’s stability in the frequency domain description is also demonstrated. More importantly, the deviation of the low wavenumber limit S(0) varies with the projection area fraction, which becomes more pronounced as the volume fraction increases, indicating that at higher volume fractions, the effective spherical model’s description approaches the actual system more closely.

Considering the ultrasound under low-frequency conditions, the “average sensing” feature of the macroscopic structure is illustrated by the consistency of S(0) in the effective model prediction of ultrasound attenuation, providing important theoretical support for the effective model.

### 4.2. Distribution Attenuation and Comparison Analysis of Multi-Model Prediction Results

**Figure 7:**
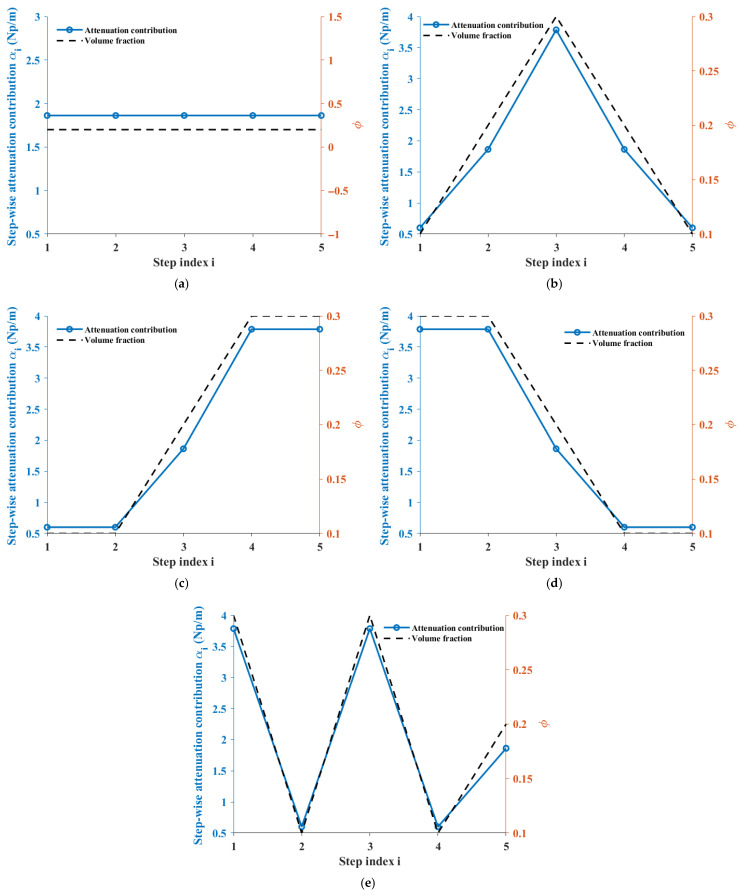
Stepwise attenuation contributions along the propagation path for five specimen configurations. The plots show incremental scattering-induced attenuation associated with successive discretized segments used in the path-integrated formulation. (**a**) Group 1; (**b**) Group 2; (**c**) Group 3; (**d**) Group 4; (**e**) Group 5. The group classifications correspond to different aggregate spatial distributions defined in the experimental design.

As shown in Figure 7a–e, the gradual attenuation contributions at five propagation step positions for five sample sets are provided, along with the corresponding distribution of aggregate volume fractions. The left axis represents the normalized attenuation contributions at each step, while the right axis shows the corresponding volume fractions. The results indicate a significant correlation between the local volume fraction distribution and the gradual attenuation behavior. The volume fractions for the five sample sets, arranged in the order from the transmitting transducer to the receiving transducer, are as follows:

(a) Group 1: *ϕ* = [0.20, 0.20, 0.20, 0.20, 0.20];
(b) Group 2: *ϕ* = [0.10, 0.20, 0.30, 0.20, 0.10];
(c) Group 3: *ϕ* = [0.10, 0.10, 0.20, 0.30, 0.30];
(d) Group 4: *ϕ* = [0.30, 0.30, 0.20, 0.10, 0.10];
(e) Group 5: *ϕ* = [0.30, 0.10, 0.30, 0.10, 0.20];

From the gradual attenuation curves, it can be seen that the attenuation contributions at each step show a highly consistent variation trend with the corresponding aggregate volume fractions. At positions with higher volume fractions, the attenuation contributions significantly increase; while at positions with lower volume fractions, the attenuation contributions decrease accordingly.

Taking Group 2 and Group 3 as examples, their volume fractions exhibit a distinct “central concentration” characteristic along the propagation path. The corresponding gradual attenuation curves show a peak near the third step, and the curve shape changes almost synchronously with the volume fraction distribution. This result indicates that the attenuation model constructed based on the path integral form can effectively capture the modulation effect of local aggregate distribution on ultrasonic attenuation, rather than just reflecting the overall average effect.

For Group 1, the volume fraction remains constant along the propagation path, and the gradual attenuation contributions remain stable, showing no significant fluctuations. This phenomenon further validates the physical consistency of the model under conditions of uniform spatial distribution.

**Figure 8:**
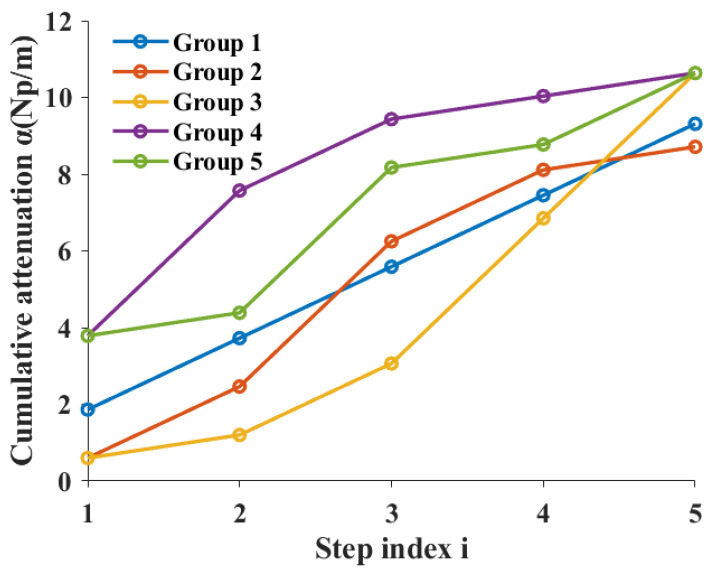
Cumulative attenuation versus propagation distance for different specimen configurations. Attenuation is obtained by integrating incremental scattering contributions from successive discretized segments, illustrating the path-dependent energy loss predicted by the proposed model. Configuration definitions correspond to the aggregate spatial arrangements specified in the experimental design.

Figure 8 shows the cumulative attenuation curves along the propagation path for the five sample sets. It can be observed that the cumulative attenuation for all samples increases monotonically with the number of propagation steps, but the rate of increase varies significantly between different groups.

For Group 4, which has higher volume fractions in the earlier part of the path, the cumulative attenuation increases rapidly in the first two steps, and then the rate of increase slows down noticeably. In contrast, Group 3, which has higher volume fractions in the later part of the path, exhibits more significant cumulative attenuation in the subsequent steps. This difference clearly reflects that attenuation is not solely determined by the total propagation length, but is closely related to the spatial distribution of the aggregate along the propagation path.

It is worth noting that, despite the different volume fraction combinations for each sample, the final cumulative attenuation levels remain within a similar range. This phenomenon provides a reasonable basis for integrating the gradual attenuation results into an equivalent propagation path attenuation coefficient in subsequent analyses.

**Figure 9:**
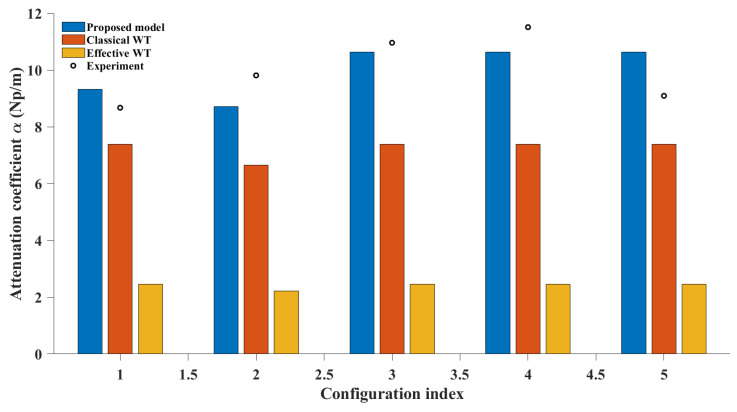
Comparison of cumulative attenuation coefficients predicted by different models and experimental measurements for five specimen configurations. Bars represent results from the proposed path-integrated model, the Classical WT model based on Rayleigh scattering, and the Effective WT model using parameterized multiple scattering formulation. Markers denote experimentally measured attenuation coefficients. The comparison illustrates relative predictive performance across configurations.

To quantitatively assess the predictive ability of different attenuation models, this study compares the cumulative attenuation coefficients obtained from the proposed model with the prediction results based on classical wave theory (Classical WT) [32] using Rayleigh scattering, and the effective wave theory (Effective WT) model [33] constructed using a parameterized multiple scattering formula, as shown in Figure 9. The models’ absolute accuracy and trend consistency are systematically evaluated using statistical indicators, including root mean square error, mean absolute error, and mean absolute percentage error, with detailed results provided in Table 4.

The statistical results show that the proposed model significantly improves the agreement with the experimental measurements, with a much lower prediction error and a more reasonable fit to the gradual attenuation along the propagation path. Compared to this, the Classical WT model exhibits significant bias, showing that the single-scattering approximation based on Rayleigh scattering is insufficient to replicate the attenuation levels in the non-uniform aggregate volume fraction distribution. The Effective WT model shows a more obvious bias, indicating that it tends to deviate from the system’s inherent trend.

From a physical mechanism perspective, the performance deficiencies of the two models are rooted in the assumption of uniform spatial scattering distribution. Both the Classical WT and Effective WT models employ a single-phase effective wave equation, and the attenuation results are mainly influenced by the overall average wavenumber:

(42) $$k_{\mathrm{eff}}=k_{0}\left[\begin{array}{c} 1+C_{s}\phi {\left(k_{0}a\right)}^{3} \end{array}\right]$$

Although this formula ensures that the attenuation contributions are strictly positive, it implicitly neglects the stepwise redistribution effect of scattering intensity along the propagation path. This leads to the local variation in volume fraction and microstructural order being averaged out, resulting in cumulative errors during the segmented integration. In contrast, the proposed model explicitly considers the spatial sequence of volume fraction configurations, allowing the cumulative attenuation to naturally exhibit the superimposed effect of stepwise contributions. This not only explains the significant reduction in error metrics but also aligns with the monotonic attenuation growth observed experimentally.

From both the statistical evidence and physical explanation, it is clear that the proposed model not only improves prediction accuracy but also provides a more reasonable theoretical framework for describing the attenuation evolution of non-uniform media, where the redistribution of scattering bodies plays a dominant role.

The following conclusions can be drawn from the results of gradual attenuation, cumulative attenuation, and multi-model comparison:

1. Ultrasonic attenuation under multi-sample serial conditions exhibits significant path dependence, with the local aggregate volume fraction distribution having a direct impact on the attenuation contributions.
2. The model based on the path integral form effectively maps local structural information to the macroscopic attenuation results, maintaining high prediction accuracy under non-uniform distribution conditions.
3. Compared to the Classical WT and Effective WT models, the proposed model significantly improves the fit to experimental results while keeping the number of parameters limited, demonstrating its applicability to practical ultrasonic testing problems in concrete.

## 5. Conclusions

This study presented a path-integrated ultrasonic attenuation modeling framework for heterogeneous concrete containing randomly distributed aggregates. By coupling a quasi-one-dimensional discretized wave equation with a modified Waterman–Truell effective medium theory, the proposed approach explicitly accounts for aggregate-induced multiple scattering and spatial non-uniformity along the propagation path. The incorporation of a Percus–Yevick structure factor and a geometric equivalence scheme for non-spherical aggregates enables a more physically consistent description of effective wave attenuation under moderate aggregate volume fractions.

Low-frequency ultrasonic transmission experiments using serially assembled concrete specimens verified the validity of the proposed framework. The experimental results demonstrated that ultrasonic attenuation exhibits pronounced path-dependent behavior, with local aggregate volume fraction directly governing stepwise attenuation contributions. Compared with classical and parameterized Waterman–Truell models, the proposed method significantly improved prediction accuracy, achieving a mean absolute percentage error of 7.29%, while maintaining clear physical interpretability.

The findings highlight that treating ultrasonic attenuation as a cumulative process governed by local structural statistics is essential for reliable sensing of heterogeneous concrete. Rather than relying solely on globally averaged parameters, the proposed path-integrated formulation enables ultrasonic measurements to be more directly linked to spatial variations in internal structure. This characteristic is particularly relevant for non-destructive evaluation and structural health monitoring of high-end concrete-based engineering structures, where material heterogeneity plays a critical role in performance and safety.

To further strengthen methodological validation and physical interpretation, future investigations will include more systematic quantitative comparisons between the modified Waterman–Truell formulation and alternative multiple scattering frameworks. In particular, sensitivity analyses on key modeling assumptions—including quasi-one-dimensional propagation, discretization scale, and equivalent-geometry representation—will be conducted to delineate their domains of validity.

In addition, broader benchmarking against numerical wave simulations and other effective medium approaches will be pursued to better quantify model robustness under varying aggregate statistics and frequency regimes. Such efforts will also enable deeper physical interpretation of attenuation mechanisms by separating contributions from local scattering strength, spatial correlation, and cumulative energy redistribution. These extensions will provide a more comprehensive assessment of the modeling assumptions highlighted in this study and further reinforce the theoretical basis of path-integrated ultrasonic attenuation prediction.

Future work will focus on extending the framework to broader frequency ranges, incorporating time-dependent material evolution, and integrating the model with advanced ultrasonic sensing configurations to support in situ monitoring of complex concrete structures under realistic service conditions.

## Figures and Tables

**Table 1:** Chemical composition (mass fraction) of P·O 42.5 cement.

Ignition Loss	SiO_2_	Al_2_O_3_	Fe_2_O_3_	CaO	MgO	SO_3_
≤4%	26.38	9.61	4.34	50.09	3.16	2.01

**Table 2:** Material quality ratio of a single mold.

Volume Fraction *ϕ*	Cement (g)	Water (g)	Aggregate (g)
0.10	376.3	150.5	78.0
0.20	334.5	133.8	156.0
0.30	292.7	117.1	234.0

**Table 3:** RMMS of p(r), g(r), S(q) and the difference in S(0).

Projected Area Fraction	p(r) RMMS	g(r) RMMS	S(q) RMMS	S(0) Difference
0.15	1.78 × 10^−3^	1.63 × 10^−1^	9.83 × 10^−2^	0.57
0.25	1.160 × 10^−3^	1.11 × 10^−1^	8.46 × 10^−2^	0.30
0.35	6.02 × 10^−4^	1.08 × 10^−1^	8.67 × 10^−2^	0.21

**Table 4:** Statistical indicator results for different models.

Model	RMSE Np/m	MAE Np/m	MAPE%
Proposed model	0.79	0.74	7.29
Classical WT	3.21	3.03	28.83
Effective WT	7.91	7.84	76.28

## Data Availability

Data are contained within the article.
